# Comparison of computed tomography image features extracted by radiomics, self-supervised learning and end-to-end deep learning for outcome prediction of oropharyngeal cancer

**DOI:** 10.1016/j.phro.2023.100502

**Published:** 2023-11-07

**Authors:** Baoqiang Ma, Jiapan Guo, Hung Chu, Lisanne V. van Dijk, Peter M.A. van Ooijen, Johannes A. Langendijk, Stefan Both, Nanna M. Sijtsema

**Affiliations:** a Department of Radiation Oncology, University of Groningen, University Medical Center Groningen, Groningen, Netherlands; b Machine Learning Lab, Data Science Center in Health (DASH), Groningen, Netherlands; c Bernoulli Institute for Mathematics, Computer Science and Artificial Intelligence , University of Groningen, Groningen, Netherlands; d Center for Information Technology, University of Groningen ,Groningen, Netherlands; e Department of Radiation Oncology, University of Texas MD Anderson Cancer Center, Houston, TX USA

**Keywords:** Oropharynx carcinoma, Radiomics, Deep learning, Self-supervised learning, Prognostic modeling

## Abstract

**Background and purpose:**

To compare the prediction performance of image features of computed tomography (CT) images extracted by radiomics, self-supervised learning and end-to-end deep learning for local control (LC), regional control (RC), locoregional control (LRC), distant metastasis-free survival (DMFS), tumor-specific survival (TSS), overall survival (OS) and disease-free survival (DFS) of oropharyngeal squamous cell carcinoma (OPSCC) patients after (chemo)radiotherapy.

**Methods and materials:**

The OPC-Radiomics dataset was used for model development and independent internal testing and the UMCG-OPC set for external testing. Image features were extracted from the Gross Tumor Volume contours of the primary tumor (GTVt) regions in CT scans when using radiomics or a self-supervised learning-based method (autoencoder). Clinical and combined (radiomics, autoencoder or end-to-end) models were built using multivariable Cox proportional-hazard analysis with clinical features only and both clinical and image features for LC, RC, LRC, DMFS, TSS, OS and DFS prediction, respectively.

**Results:**

In the internal test set, combined autoencoder models performed better than clinical models and combined radiomics models for LC, RC, LRC, DMFS, TSS and DFS prediction (largest improvements in C-index: 0.91 vs. 0.76 in RC and 0.74 vs. 0.60 in DMFS). In the external test set, combined radiomics models performed better than clinical and combined autoencoder models for all endpoints (largest improvements in LC, 0.82 vs. 0.71). Furthermore, combined models performed better in risk stratification than clinical models and showed good calibration for most endpoints.

**Conclusions:**

Image features extracted using self-supervised learning showed best internal prediction performance while radiomics features have better external generalizability.

## Highlights

• CT image features were extracted based on three methods.
• Image features were used in multi outcome prediction models of oropharyngeal squamous cell carcinoma.
• Self-supervised learning extracted Image features showed best internal prediction.
• Radiomics features have better external generalizability.

## 1 Introduction

Head and neck cancer (HNC) is a common cancer type worldwide and is commonly treated with surgery, (chemo)radiotherapy, or both. Oropharyngeal squamous cell carcinoma (OPSCC), an important type of HNC, includes human papillomavirus (HPV) related (HPV + ) and HPV-unrelated (HPV-) tumors, which are mostly related to cigarette and alcohol use. 5-year overall survival (OS) rate in patients with HPV + tumors is generally better (75 %-80 %) than in those with HPV-negative tumors (45 %-50 %) [1]. To allow for more individualized treatment choices in the future, it is necessary that outcome prediction models with good performance become available for different endpoints like local control (LC), regional control (RC), distant metastasis free survival (DMFS) and OS.

Clinical parameters such as HPV-status, age, gender, T-stage, N-stage and smoking status have been identified as prognostic factors of OS [2], [3], [4], [5], [6], [7], [8], [9], [10], [11], [12], progression-free survival (PFS) [7], [8], [11] and locoregional control (LRC) [3] for OPSCC patients. The risk stratification system for OS in OPSCC patients based on HPV-status, pack years of smoking, tumor and nodal stages proposed by Ang et al. [2] has a good performance and is commonly used clinically. However, the availability and performance of clinical models for other endpoints like local and regional control is still limited [3], [13]. Some studies showed that the addition of radiomic features extracted from MRI [3] or PET/CT [14], [15], [16], [17] improved the performance of prediction models for LRC and OS compared to that of clinical models. However, these hand-crafted radiomics features are limited describing the inherent characteristics of tumors.

Compared to radiomics, convolutional neural networks (CNNs) can extract more representative and descriptive image features and have been successfully applied in various tasks such as image synthesis [18], [19], [20], super-resolution [21], [22] and segmentation [23], [24]. Recently, researchers have applied CNNs in radiotherapy applications including automatic segmentation [25], [26], treatment planning [27] and outcome prediction [28], [29], [30]. For the outcome prediction of OPSCC, Fujima et. al. used CNNs to extract PET image features to predict local treatment outcomes [31]. Moreover, Cheng et. al. proposed a fully automatic tumor segmentation and OS prediction tool of OPSCC based on CNNs [32]. Naser et. al. applied a DenseNet based method to extract features from CT, PET, (gross tumor volumes of the primary tumor) GTVt and clinical data together to predict PFS of OPSCC [33]. Our previous studies extracted features from CT, PET and GTVt using self-supervised or end-to-end learning based methods and built deep learning models based on clinical and image features together for outcome prediction [34], [35], [36]. However, these works relied on PET images which are less available than planning CTs.

Based on CT only, Diamant et. al. built 2D CNN models with an input of 2D central tumor slice of pretreatment CTs [37], and achieved better prediction than previous radiomics models [38] in the prediction of locoregional failure, distant metastasis (DM) and OS of HNC. Lombardo et. al extended this 2D CNN to 3D for time-to-event DM prediction [39]. Their 3D CNNs generally obtained good C-index values of around 0.80 in the validation sets and two of three independent test sets. Wang et. al proposed a 3D residual block-based model and achieved C-indexes of 0.77 and 0.64 for DM and OS prediction when inputting the 3D CT volume of the GTV (gross tumor volume) region [40]. The above studies included a comparison of radiomics models and end-to-end deep learning models. However, they investigated a limited set of endpoints and did not always compare the radiomics and CNN models to prediction models using clinical features only. In this study, we aimed to extract tumor image features from the pretreatment CT and investigated whether these image features can improve the performance of clinical parameters-based outcome prediction models for OPSCC patients. The predicted abilities of CT image features extracted by radiomics, self-supervised learning (autoencoder) and end-to-end deep learning, respectively, were investigated and compared for the prediction of local control (LC), regional control (RC), LRC, distant metastasis-free survival (DMFS), tumor-specific survival (TSS), OS and DFS (disease-free survival).

## 2 Material and methods

**Fig. 1:**
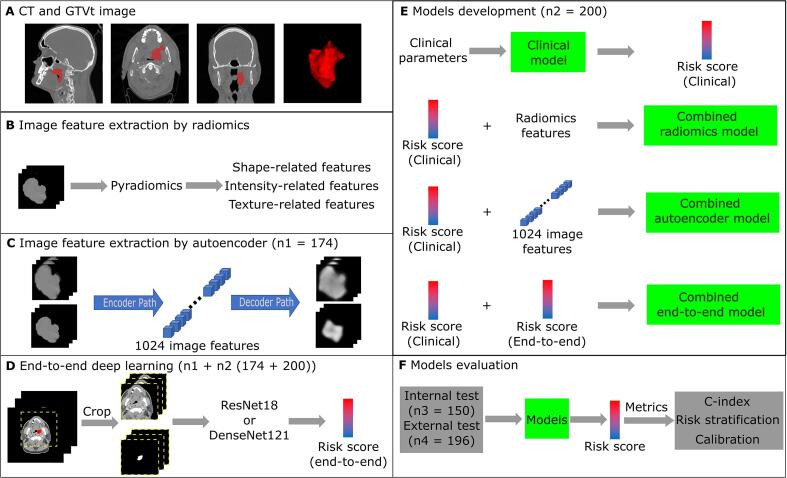
Flowchart of feature extraction, model training and evaluation of OPSCC outcome prediction models. **A** Example of planning CT scan in three directions and the corresponding gross primary tumor volume (GTVt). **B** Image feature extraction by radiomics. **C** Autoencoder for extracting image features from CT tumor images. **D** End-to-end models inputting CT and GTVt for risk score prediction. **E** Models development using Cox regression. **F** Models evaluation.

The flowchart of image feature extraction, model training and evaluation is illustrated in Fig. 1.

### 2.1 Patient demographics, imaging data and endpoints

The cohort that was used for model development is the OPC-Radiomics dataset [41] which includes 606 OPSCC patients who received (chemo-) radiotherapy at Princess Margaret Cancer Centre. From this set, 524 patients with planning CT scans (around 75 % were contrast-enhanced) and manually delineated gross tumor volumes of the primary tumor (GTVt) available were randomly split into subsets of n1 = 174, n2 = 200 and n3 = 150 patients that were used for the autoencoder training, building outcome prediction models and independent internal testing, respectively. A detailed description of the OPC-Radiomics set is publicly available at TCIA [41]. The external test (n4 = 197) was performed on the UMCG-OPC dataset (detailed description in Supplementary 1) comprising 197 OPSCC patients. All OPC-Radiomics and UMCG-OPC patients were without distant metastasis at diagnosis.

Clinical candidate predictors and outcome endpoints are explained in Supplementary 2.

### 2.2 **Image feature extraction by radiomics**

In Fig. 1B, 110 radiomics features were extracted for each patient (details in Supplementary 3).

### 2.3 Image feature extraction by autoencoder

Image features were extracted from the GTVt volume in the CT by the pyramid autoencoder, which is a self-supervised learning-based CNN. The input has two channels which were obtained by the method described in Supplementary 4. The autoencoder (Fig. 1C) consists of an encoder path for extracting 1024 representative image features from the input 3D CT tumor images and a decoder aiming to reconstruct the input images from the image features. The detailed description of the architecture (Figure S1) and training strategies of the autoencoder are displayed in Supplementary 4. Each autoencoder extracted image feature was normalized by the maximum and minimum values of this image feature in the training cohort.

### 2.4 End-to-end deep learning

An end-to-end deep learning method was built to directly predict the risk score of each endpoint (Fig. 1D, detailed description in Supplementary 5)._

### 2.5 Outcome prediction models development

From Fig. 1E, clinical models were first built using multivariable Cox proportional hazard regression analysis for the prediction of each outcome endpoint. The forward selection process was repeated 1000 times using bootstrapping samples in the patients from n1 and n2. In each time of forward selection, the C-index increase was the criteria and the largest number of selected features was set to 5. After each forward selection, only significant predictors (p < 0.05 of the Wald test) were finally selected. After 1000 times of forward selection, the selected frequency of features in all 1000 times were ranked. Then, the most frequently selected clinical features which had a significant contribution (p < 0.05 of the Wald test) when used together in the clinical model were included in the final clinical model.

For the combined radiomics and autoencoder models, the linear predictor of the clinical model was considered as one feature together with the radiomics features or the 1024 image features extracted by the autoencoder and were used to perform the same multivariable Cox proportional hazard regression analysis, as shown for the clinical model above. Then, the most frequently selected features were used to build combined radiomics and autoencoder models.

The two predicted risk scores from the clinical model and the end-to-end deep learning model were used to build combined end-to-end models for each endpoint. The models implementation code can be found in https://github.com/baoqiangmaUMCG/Ctfeatures-Outcome-Prediction.

### 2.6 Model performance evaluation

The concordance index (C-index) [95 % confidence interval (CI)] was first applied to evaluate the discriminative ability, with the z-test to compare C-index differences between models. In detail, the z-test compared the difference of 1000 C-indexes calculated on 1000 bootstrapping samples. Then, patients were divided into a high-risk group (hazard value > the median hazard of the training set) and a low-risk group (hazard value <= the median) for each endpoint, with the log-rank tests [42] to determine the significant differences of Kaplan-Meier (KM) curves between groups for each outcome. Additionally, the calibration ability of the models was determined by comparing the curves of outcome rates predicted by the combined models with the KM curves (95 % CI) of the observed outcomes within 5-year follow-up. The predicted curves of each outcome endpoint such as OS were obtained by averaging the predicted OS curves of each patient in the test cohorts. Finally, we compared actual and predicted 2-year outcomes rate with the Hosmer Lemeshow (HS) test for evaluating goodness of fit and calculated the calibration slope and intercept. A two-tailed p-value < 0.05 was considered significant. A significant HS test indicates a poor calibration.

## 3 Results

Table S1 and Table S2 displayed the differences between OPC-Radiomics and UMCG OPC in clinical data and outcomes (details in Supplementary 6).

**Fig. 2:**
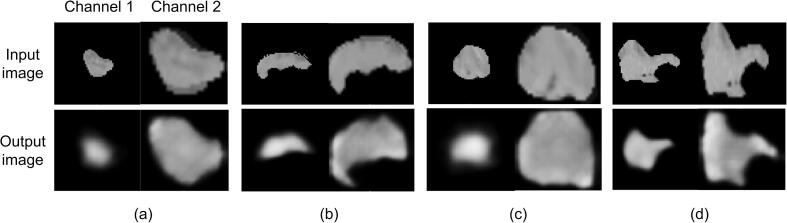
Four examples of input and output CT tumor images of autoencoders.

Fig. 2 displays four examples of the input and output (reconstructed by the autoencoder) CT tumor images. The shapes and CT intensities of tumors were generally reconstructed by the autoencoder, which means that the extracted image features are representative and descriptive for the tumor images. Additionally, the autoencoder achieved the mean squared error of 0.037 and 0.020, and the Structural Similarity [43] of 0.704 and 0.706, in the internal and external tests, respectively.

The C-index values of clinical, combined autoencoder and combined radiomics models are shown in Table 1, in which the clinical models achieved high C-index values in the training set (between 0.67 and 0.81), the independent internal test set (from 0.60 to 0.76) and the external test set (from 0.67 to 0.80). The combined autoencoder models obtained higher C-index values than clinical models for all outcomes in the training and independent internal test sets, with the largest C-index improvements in RC (from 0.76 to 0.91), DMFS (from 0.60 to 0.74) and the smallest improvement < 0.01 for OS in the independent internal test set. The C-index of 0.74 for DMFS is comparable to 0.69, the best test C-index in OPC-radiomics set, achieved by the CNN models proposed by Lombardo et. al [39]. In the external test set, combined autoencoder models achieved higher C-index values in LC, LRC, TSS (C-index improvement < 0.01), OS (C-index improvement < 0.01) and DFS than clinical models with the highest C-index improvement from 0.71 to 0.76 in LC. The combined radiomics models achieved higher C-index values than clinical models for all endpoints in the training and external test sets, and for LC, RC, LRC, DMFS, OS (improvement < 0.01) in the internal test set. After comparison of the three models, the combined autoencoder models obtained significant highest C-indexes for all endpoints except OS in the internal test set while the combined radiomics models had the highest C-indexes for all endpoints in the external test set. As displayed in Table S4, the combined end-to-end models did not achieve significantly higher C-index values for most endpoints in both internal and external test sets while it did in the training set. The combined models generally keep their better performance than clinical models for HPV positive and negative patients, respectively (Supplementary 8).

**Table 1:** C-index [95% confidence interval] results of clinical and combined autoencoder or radiomics models.

	Training set			Internal test set			External test set		
	Clinical model	Combined autoencoder model	Combined radiomics model	Clinical model	Combined autoencoder model	Combined radiomics model	Clinical model	Combined autoencoder model	Combined radiomics model
LC	0.81[0.73,0.89]	**0.84** **[0.75,0.92]**	0.84[0.74,0.92]	0.73[0.55,0.85]	**0.79** [0.59,0.93]*	0.79[0.57, 0.92]	0.71[0.56,0.86]	0.76[0.64,0.86]	**0.82** [0.72,0.90]*
RC	0.74[0.62,0.86]	**0.86** [0.76,0.93]*	0.79[0.67,0.91]	0.76[0.52,0.96]	**0.91** [0.82,0.98]*	0.84[0.70,0.97]	0.70[0.57,0.81]	0.65[0.52,0.76]	**0.76** [0.67,0.85]*
LRC	0.71[0.60,0.81]	**0.81** [0.74,0.88]*	0.74[0.63,0.85]	0.72[0.59,0.85]	**0.78** [0.63,0.91]*	0.77[0.66,0.88]	0.71[0.62,0.79]	0.72[0.64,0.81]	**0.75** [0.66,0.83]*
DMFS	0.67[0.58,0.75]	**0.75** [0.66,0.83]*	0.73[0.65,0.81]	0.60[0.49,0.72]	**0.74** [0.63,0.84]*	0.67[0.55,0.78]	0.67[0.58,0.76]	0.64[0.50,0.78]	**0.72** [0.61,082]*
TSS	0.76[0.67,0.83]	**0.79** [0.72,0.85]*	0.77[0.70,0.84]	0.70[0.59,0.80]	**0.71** [0.60,0.81]*	0.70[0.59,0.80]	0.77[0.68,0.85]	0.77[0.68,0.85]	**0.79** [0.69, 0.87]*
OS	0.73[0.68,0.78]	**0.76** [0.71,0.80]*	0.75[0.70, 0.79]	0.72[0.64,0.78]	0.72[0.64,0.78]	**0.72** **[0.64, 0.78]**	0.80[0.74,0.86]	0.80[0.74,0.86]	**0.81** [0.75,0.87]*
DFS	0.71[0.66,0.76]	**0.74** **[0.69,0.79]**	0.74[0.68,0.79]	0.72[0.65,0.78]	**0.73** [0.66,0.80]*	0.72[0.64,0.78]	0.71[0.64,0.79]	0.73[0.66,0.80]	**0.77** [0.71,0.83]*

The underlined C-indexes are slightly higher (C-index improvement < 0.01) than that of the other model in the same dataset. *: Significant difference of C-indexes between the clinical model and combined model (p-Value < 0.05 by z-test).

**Fig. 3:**
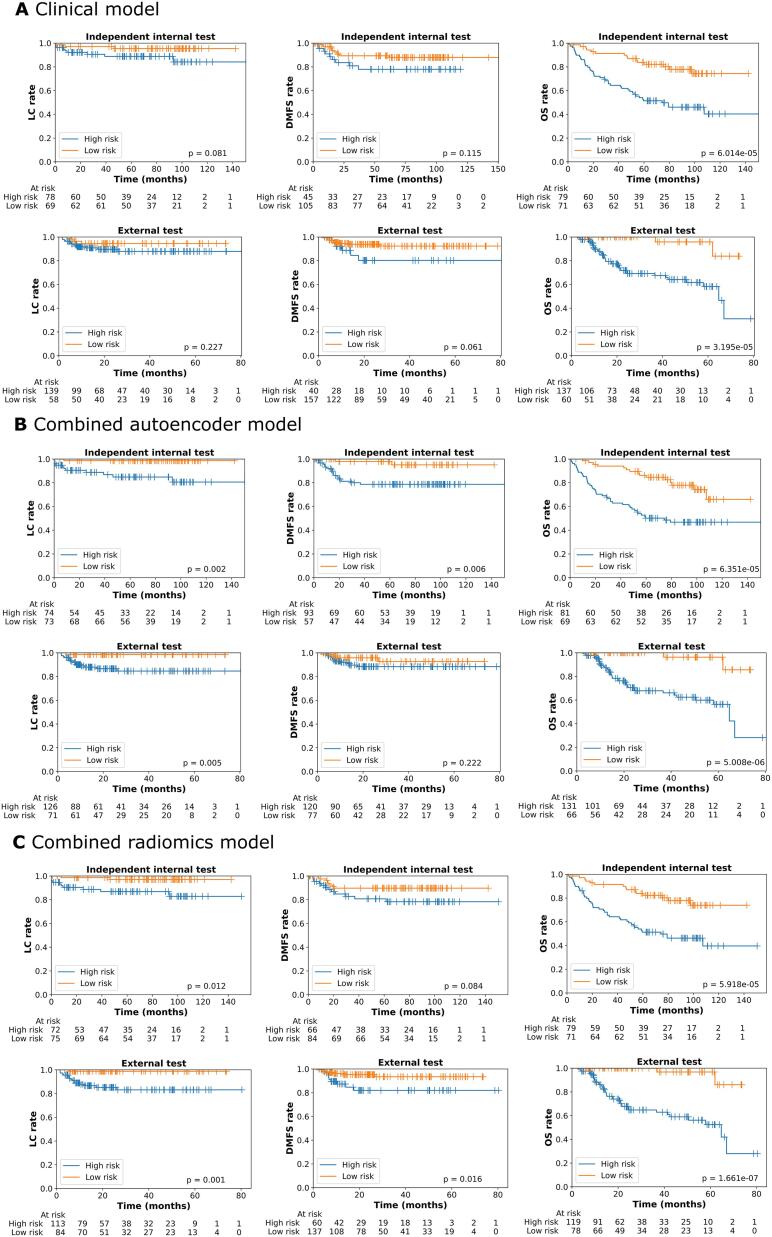
KM curves of high (hazard values > median) and low (hazard values <= median) risk groups of LC, DMFS and OS in the independent internal and external test sets stratified by clinical models **(A)** and combined autoencoder models **(B)** and combined radiomics models **(C)**. P-values < 0.05 means significant difference between two risk groups.

Fig. 3 and Figure S2 show the KM curves of high and low risk groups stratified by the clinical (A), combined autoencoder models (B) and combine radiomics models (C) for LC, DMFS and OS, and all endpoints, respectively. The p-values of the log rank tests show that clinical models can stratify patients with significant differences for (LRC, TSS, OS and DFS) and (RC, LRC, TSS, OS and DFS) in the internal and external test sets, respectively. Combined autoencoder models and combined radiomics models showed significant differences for all endpoints except for DMFS in the external test set and except for DMFS in the internal test set, respectively.

**Fig. 4:**
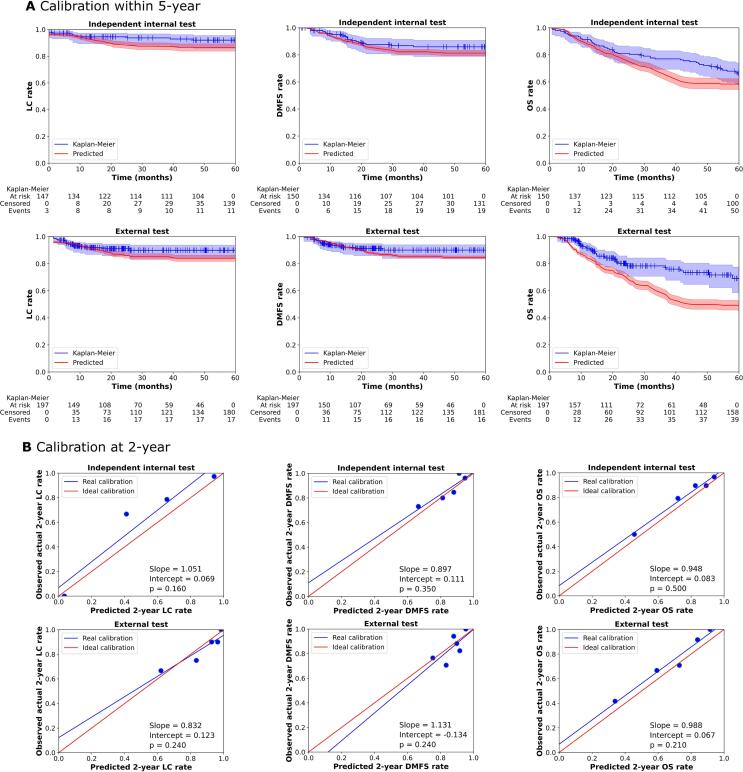
Calibration performance of combined autoencoder models for LC, DMFS and DFS in the independent internal and external test sets in the (**A**) within 5-year (**B**) at 2-year. p-Values were from HS tests. Slope and intercept belong to real calibration line. The figures in (A) showed the comparison of real Kaplan-Meier curves and the predicted outcome rates curves by combined models within 5-year follow-up.

Fig. 4A and Figure S3A show the calibration curves of the combined autoencoder models. The 95 % CIs of predicted curves and the actual KM curves overlap for LC, DMFS, TSS, OS and DFS in the internal test set and for LC, RC, LRC, DMFS and DFS in the external test set within a 2 year follow up period. According to the p-values in Fig. 4B and S3B, combined autoencoder models showed good calibration performance (p > 0.05 by HS test) for 2-year LC, DMFS, TSS, OS and DFS in the internal test set and for all 2-year endpoints except RC in the external test set. Additionally, the obtained real 2-year calibration lines are good (slope within [0.8, 1.2] and intercept within [-0.2, 0.2]) for all endpoints except RC in the external test. Curves of combined radiomics models are descripted in Supplementary 9.

## 4 Discussion

This study investigated and compared the added value of CT-image features extracted by radiomics, self-supervised learning (autoencoder) and end-to-end deep learning, respectively in the prediction of LC, RC, LRC, DMFS, TSS, OS and DFS of OPSCC patients treated with (chemo)radiotherapy. Combined autoencoder models which combined self-supervised learning extracted image features with a linear predictor from a clinical prediction model showed better discriminative performance for most outcomes in the internal test set than combined radiomics models. However, combined radiomics models showed best predictive performance for all endpoints in the external test set, which shows the better generalizability of radiomics features than autoencoder extracted features. Combined end-to-end models did not perform better than combined autoencoder models for most endpoints in both the internal and external test sets.

Compared with clinical models, combined autoencoder models achieved higher C-index values for all endpoints in the training and internal test sets as shown in Table 1, which indicates that the self-supervised learning (SLL) extracted high-level CT image features provide complementary information for outcome prediction. Other studies identified SLL extracted image features that were predictive for ovarian cancer [44], colorectal cancer [45] and gastric cancer [46]. In the external test set our combined autoencoder models still performed generally better than clinical models. However, the C-index differences between combined autoencoder and clinical models were small (<0.01) for OS in the internal test set and TSS and OS in the external test set (Table 1). This may be due to that the clinical models using four and five predictors (Table S3) for TSS, and OS prediction, respectively, already achieved a satisfactory performance and image features could not add much new predictive information.

Although combined radiomics models achieved higher C-indexes than clinical models for most endpoints in the internal test set, they were still worse than combined autoencoder models (Table 1). This is most probably because the autoencoder can extract more comprehensive and representative features which can provide more information for outcome prediction than radiomics features. However, in the external test set, combined radiomics models showed significantly higher C-indexes than the combined autoencoder models in all endpoints, which demonstrates that the selected radiomics features (Table S3) have better external generalizability than autoencoder extracted features. From Table S3, we can observe that the combined radiomics models for LC, RC, DMFS, OS and DFS prediction contained mainly shape features that are possibly less affected by differences between CT equipment and scan protocols between institutions than autoencoder extracted features. The combined end-to-end models only performed better than the autoencoder combined models for most endpoints in the training set and not in the test sets. This indicates that there was an overfitting problem.

In Fig. 3 and Figure S2, combined (autoencoder or radiomics) models stratified patients into high and low risk groups with significant differences (p < 0.05) for LC, RC and DMFS in the independent internal test set and LC in the external test sets while clinical models did not. This demonstrates that combined models could be more powerful in identifying high- and low- risk patients for individualized treatment by adding image features extracted by autoencoder or radiomics. Additionally, we found that the combined radiomics model could achieve a significant risk stratification for DMFS in the external test set while the combined autoencoder model could not. This may demonstrate that the shape feature: original_shape_MinorAxisLength selected for DMFS prediction (Table S3) is more stable than autoencoder extracted features when using it externally.

Combined autoencoder models showed good calibration for most endpoints in both test sets. For example, the combined autoencoder model achieved a good calibration slope of 0.988 and intercept of 0.067 for 2-year OS rate prediction (Fig. 4B) in the external test set as well as higher C-index of 0.80 (Table 1) and better OS risk stratification (Fig. 3) than the clinical model. Similarly, combined autoencoder model showed good calibration (Fig. 4A and 4B), better C-index values (Table 1) and better risk stratification (Fig. 3) than the clinical model for LC rate in both internal and external test sets. Thus, combined autoencoder models are highly effective for LC and OS prediction even in the external test set. This makes the combined autoencoder models promising for clinical tools for selecting patients for personalized treatments. However, in Fig. 4 and Figure S2, the 2-year calibration curves of combined autoencoder models for RC and LRC in the internal test set and RC in the external test set showed a p-value < 0.05 in the HS test indicating a poor calibration. This may be due to the low numbers of 2-year events (RR: 5 and 18 in the internal and external test sets, respectively and LRC: 10 in the internal test set). Combined radiomics models generally showed worse internal test calibration (Supplementary 9).

Additionally, we found that the tumor-volume, a radiomics feature, is not highly related to linear predictors of our combined models, and our results support previous studies [47] that deep learning features are more predictive internally and radiomics features are more stable externally (Supplementary 10). The limitations are shown in Supplementary 11.

In conclusion, we compared the abilities of CT image features extracted by radiomics, self-supervised learning and end-to-end deep learning, respectively, in improving the performance of clinical data-based prediction models for most outcomes in oropharyngeal squamous cell carcinoma patients. Self-supervised extracted features showed better predictive performance in the internal test set while radiomics features showed better generalizability when being used in the external dataset.

## CRediT authorship contribution statement

**Baoqiang Ma:** Conceptualization, Formal analysis, Investigation, Methodology, Visualization, Writing – original draft, Writing – review & editing. **Jiapan Guo:** Conceptualization, Methodology, Supervision, Writing – review & editing. **Hung Chu:** Methodology. **Lisanne V. van Dijk:** Supervision, Writing – review & editing. **Peter M.A. van Ooijen:** Supervision, Writing – review & editing. **Johannes A. Langendijk:** Data curation, Funding acquisition, Writing – review & editing. **Stefan Both:** Supervision, Writing – review & editing. **Nanna M. Sijtsema:** Project administration, Supervision, Writing – review & editing.

## Declaration of Competing Interest

The authors declare that they have no known competing financial interests or personal relationships that could have appeared to influence the work reported in this paper.
