# Supplementary material for: Comparison of computed tomography image features extracted by radiomics, self-supervised learning and end-to-end deep learning for outcome prediction of oropharyngeal cancer
Source: Phys Imaging Radiat Oncol. 2023 Nov 7;28:100502. doi: 10.1016/j.phro.2023.100502 (PMC10663809; doi:10.1016/j.phro.2023.100502)
Supplement: Supplementary data 1 [file mmc1.docx]

**Supplementary**

1. **Detailed description of UMCG-OPC set**

The UMCG-OPC dataset comprising 197 OPSCC patients who were treated with (chemo-) radiotherapy at the University Medical Center Groningen between July 2014 and December 2020. Patients with (1) a history of a neck dissection , (2) multiple primary tumors, (3) no contrast-enhanced planning CT scan, (4) no GTVt contour , or (5) no information about the HPV-status, were excluded. Finally, 197 patients were collected with contrast-enhanced planning CT scan (Somatom Sensation Open, Siemens, Forchheim, Germany; voxel size: 1.0×1.0×2.0 mm; scan voltage: 120 kV; and convolution kernel: B30) and GTVt delineated by an experienced radiation oncologist. UICC-AJCC 7th edition staging system and p16 immunohistochemistry were applied to stage patients and detect HPV-status, respectively. Patients were treated with definitive three-dimensional conformal radiotherapy (3D-CRT), intensity modulated radiotherapy (IMRT) or volumetric modulated arc therapy (VMAT) with a total dose of 70 Gy in 2 Gy fractions in 6–7 weeks, with/without chemotherapy. After 2018 some the patients were treated with intensity modulated proton radiotherapy (IMPT). Patients had follow-up appointments every 3 months in the first year after treatment and then every 6 months. Follow up information of UMCG OPSCC was available till December 2020. All UMCG patients were consecutively included in a data registration program as part of routine clinical practice, with prospective assessment of patient, tumor, and treatment characteristics, as well as radiation induced toxicities (Clinical trials NCT02435576). Since the Dutch Medical Research Involving Human Subjects Act is not applicable to data collection as part of routine clinical practice, the requirement of informed consent was waived by the ethics committee.

1. **Definitions of events and time-to-event of outcome endpoints**

Clinical candidate predictors included age, gender (female vs. male), WHO performance status (PS) (1-3 vs. 0), HPV-status (p16 positive vs. p16 negative), smoking status (current smoking vs. past-smoking vs. never smoking), T-stage (T4 vs. T1-3) and N-stage (N3 vs. N2 vs. N0-N1). Patients whose HPV-status was unknown were considered positive in the OPC-Radiomics set because the outcomes of HPV unknown patients were observed being more similar to that of HPV positive patients. The prognostic outcome endpoints were local control (LC), regional control (RC), locoregional control (LRC), distant metastasis-free survival (DMFS), tumor-specific survival (TSS), overall survival (OS) and disease-free survival (DFS), with events and time-to-event defined in Supplementary 2. The events of LC, RC and LRC were defined as residual or recurrent disease at the primary site, the regional nodes and the primary site or regional nodes, respectively. Distant metastasis was the event of DMFS. The events of OS and TSS were death caused by any reasons and death caused by the tumor, respectively. The events of DFS included all the events mentioned above. The time interval between the start of the treatment and the date of event occurrence is the time to event. Patients without event occurrence were marked as censored at the last date of follow-up.

1. **Image feature extraction by radiomics**

As shown in Figure 1B, 16 shape-related (3D), 19 intensity-related (First Order Statistics) and 75 texture-related features (Gray Level Cooccurence Matrix, Gray Level Run Length Matrix, Gray Level Size Zone Matrix, Neighbouring Gray Tone Difference Matrix and Gray Level Dependence Matrix) were extracted from each GTVt tumor region in CT using Pyradiomics v3.0.1 package (https://pyradiomics.readthedocs.io/en/latest/). The applied bin size for CT was 25. The CT interpolation method, image resolution after interpolation and texture matrices aggregation method were set to Linear interpolation, $1.0\times1.0\times1.0 mm3$ and averaging, respectively.

1. **Detailed description of the architecture and training strategies of the autoencoder**

**
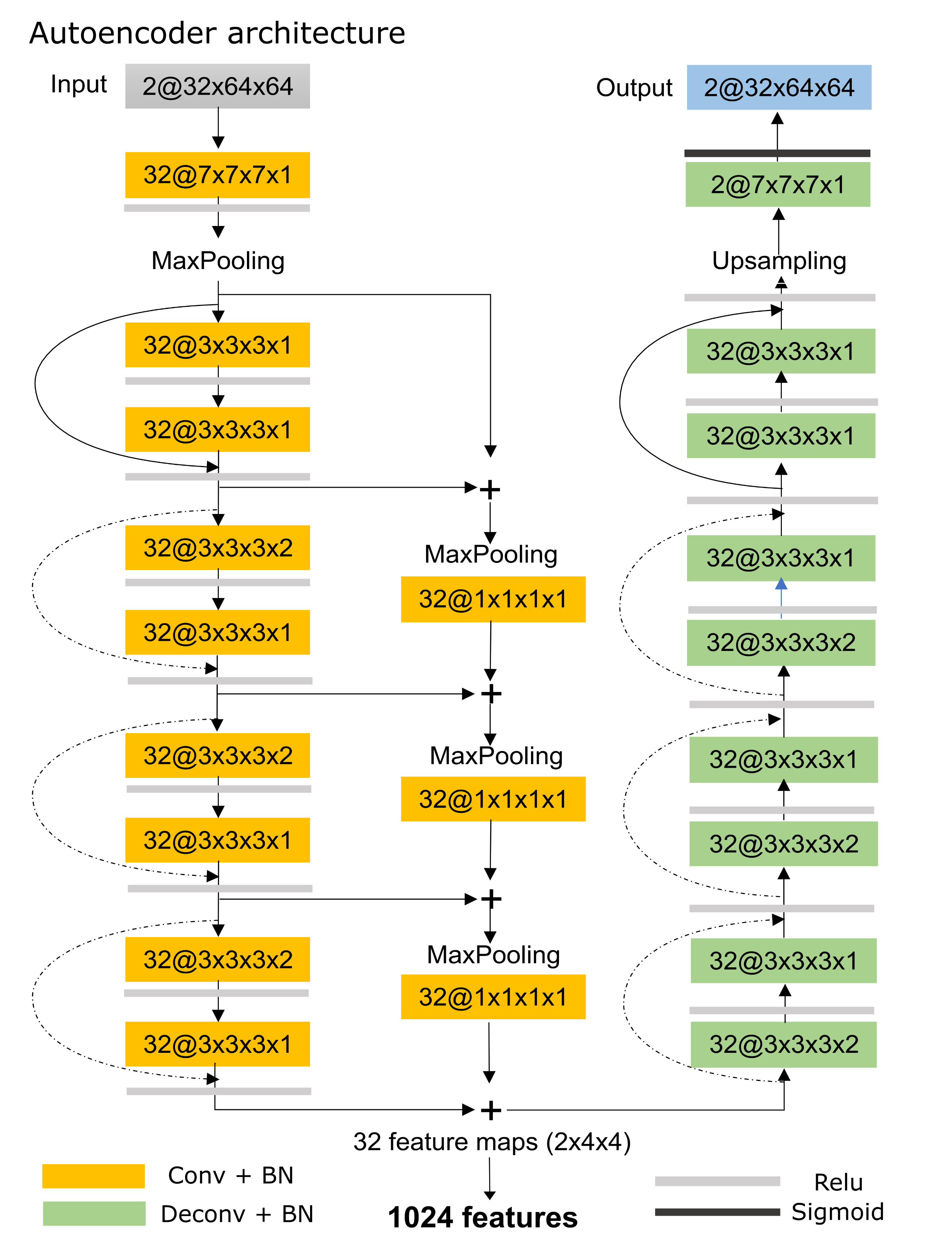
**

Figure S1. The architecture of the autoencoder. Conv: convolutional layer; Deconv: deconvolutional layer; BN: batch normalization layer; Relu: Relu activation function; Sigmoid: Sigmoid activation function; N@H×W×D×S represents the 3D convolutional or deconvolutional kernel numbers (N), size in three directions (H×W×D) and stride in three directions (S), respectively. The strides of all MaxPooling layers and the Upsampling layer are 2. Solid curves mean direct residual connection and Dash curves mean first downsampling input then residual connection.

The autoencoder was trained using patients from n1 continuously to obtain increasing similarity between the input and the output tumor image. After training, the extracted image features were expected to be highly representative of various image characteristics such as intensity, shape, and texture of the tumor image, which makes them potential predictors of prognostic outcomes of OPSCC.

Two image input channels were used. The images were generated by using two different cropping methods for the GTVt and the planning CT: (1) GTVt center cropping with the size of 32×64×64, resulting in images with the original resolution (2×1×1 mm3) which could miss part of the tumor volume in case of very large tumors; (2) GTVt border cropping and then resizing to the size of 32×64×64 resulting in images with variable resolution that always contain the whole tumor providing tumor shape and border information.

The autoencoder was built upon 3D residual convolutional and deconvolutional blocks with an additional pyramid architecture between convolutional blocks. The convolution or deconvolution kernel number ($N$), kernel size in three directions ($H\times W\times D$) and the strides ($S$) are shown as $N@H\times W\times D\times S$ in Figure S1. The encoder path was composed of one convolutional layer, a maxpooling layer, 4 continuous convolutional residual blocks and the pyramid architecture combining image features of different residual blocks. The strides of all maxpooling layers are 2. 32 latent feature maps with the size of $2\times4\times4$ were obtained at the end of the encoder. These features maps were reshaped to one vector with 1024 image features. The decoder consists of four deconvolution residual blocks, an upsampling layer with a sampling factor of 2 and a single deconvolutional layer. All convolution or deconvolutional layers were followed by a batch normalization layer. A Relu function followed either convolutional and deconvolutional layers or the end of residual blocks as shown in Figure S1.

The loss function of autoencoder training included L1 loss, mean square error (MSE) and structural similarity (SSIM). L1 loss is defined as:

$L_{1}=\left\| A_{\left( x \right)}-x \right\|_{1}$ (1)

Where $x$ is the input image and $A_{\left( x \right)}$ is the reconstructed image by autoencoder $A$.

The MSE can be written as:

$L_{MSE}=\left\| A_{\left( x \right)}-x \right\|_{2}$ (2)

The definition of SSIM can be found in [1].

Finally, the combined loss function we used is:

$L_{combined}= L_{1}+ L_{MSE}+0.5 L_{SSIM}$ (3)

The random rotations (-45 to 45 degrees) and randomly vertical and horizontal flipping were used to augment the training set for avoid over-fitting. The autoencoder was trained using Adam optimizer with a learning rate of 0.001 and a batch size of 6. The total training epoch was set to 80. The training was ran using PyTorch 1.6.0 on one Tesla V100 GPU.

1. **End-to-end deep learning**

An end-to-end deep learning method based on 3D ResNet18 [2] or DenseNet121 [3] was built to directly predict the risk score of each endpoint. For obtaining the input of these models, original CT and GTVt images were first resampled to $2 \times2\times2$ mm3 and then were cropped centering at the center of GTVt (by the size of $90 \times90\times92$). The cropped CT and GTVp were used as two channels of the input volume of models. The models were trained using a negative cox-likelihood loss [4] and other settings same with those in our previous study [5] which used the end-to-end deep model for outcome prediction of OPC. Five-fold cross validation were applied in the training set (n1 + n2) and the final risk prediction of each patient is the average ensemble of predictions of five models.

1. **Difference between OPC-Radiomics and UMCG OPC**

The statistics of clinical parameters in Table S1 displayed significantly higher proportions of females, current smokers, HPV-negative and T4-patients in the UMCG-OPC cohort (32.1%, 49.2%, 40.2% and 45.2%, respectively) than that in OPC-Radiomics cohort (19.3%, 32.3%, 23.9% and 21.9%, respectively). Table S2 shows that the UMCG-OPC set has both shorter time-to-event and follow up and a lower proportion of events for all endpoints than the OPC-Radiomics set except RR.

Table S1: Baseline characteristics of the patients in the OPC-Radiomics and UMCG-OPC sets.

|  | OPC-Radiomics |  | UMCG-OPC |  |  |
| --- | --- | --- | --- | --- | --- |
| Characteristics | N=524 | % | N=197 | % | p-Value |
| **Age at diagn. (M** *±* **std, years)**  **Gender** | 61*±*10 |  | 62*±*9 |  | 0.292*^a^*  <0.001 *^b^* |
| Male | 423 | 80.7% | 133 | 67.5% |  |
| Female | 101 | 19.3% | 64 | 32.5% |  |
| **WHO PS** |  |  |  |  | 0.134*^b^* |
| 0 | 331 | 63.2% | 145 | 73.6% |  |
| 1 | 135 | 25.8% | 40 | 20.3% |  |
| 2 | 44 | 8.4% | 10 | 5.0% |  |
| 3 | 9 | 1.7% | 2 | 1.0% |  |
| 4 | 1 | 0.0% | 0 | 0.0% |  |
| **Smoking status** |  |  |  |  | <0.001*^b^* |
| Current | 169 | 32.3% | 84 | 42.6% |  |
| Ex-smoker | 209 | 39.9% | 84 | 42.6% |  |
| Non-smoker | 146 | 27.9% | 29 | 14.7% |  |
| **HPV-status** |  |  |  |  | <0.001*^b^* |
| Negative | 125 | 23.9% | 95 | 48.2% |  |
| Positive | 305 | 58.2% | 102 | 51.8% |  |
| Unknown | 94 | 17.9% | 0 | 0.0% |  |
| **T-stage** |  |  |  |  | <0.001*^b^* |
| T1 | 64 | 12.2% | 36 | 18.2% |  |
| T2 | 170 | 32.4% | 44 | 22.3% |  |
| T3 | 175 | 33.4% | 20 | 10.2% |  |
| T4 | 115 | 21.9% | 97 | 49.2% |  |
| **N-stage** |  |  |  |  | 0.111*^b^* |
| N0 | 90 | 17.1% | 28 | 14.2% |  |
| N1 | 52 | 9.9% | 28 | 14.2% |  |
| N2 | 349 | 66.6% | 135 | 68.5% |  |
| N3 | 33 | 6.3% | 6 | 3.0% |  |

Abbreviations: T=tumor; N=lymph node; WHO PS=World Health Organization performance status; HPV=human papilloma virus status. a p-Value was calculated using z-test. b p-Value was calculated using the chi-square test.

Table S2. The median time-to-event [interquartile range], events number (percentage) and median follow up [interquartile range] in OPC-Radiomics and UMCG-OPC sets.

|  | OPC-Radiomics | | | UMCG-OPC | | |
| --- | --- | --- | --- | --- | --- | --- |
|  | Median time-to-event (Months) | Events number | Median follow up (Months) | Median time-to-event (Months) | Events number | Median follow up (Months) |
| LR | 7.7 [0.0, 20.2] | 62 (11.8%) | 69.2[26.0,100.6] | 5.6[3.0,7.9] | 17(8.6%) | 26.1[12.0,61.7] |
| RR | 9.1 [0.0,15.8] | 37 (7.1%) | 69.2[26.9,100.6] | 9.5[5.4,16.1] | 20 (10.1%) | 25.8[11.4,61.7] |
| LRR | 7.7 [0.0,20.2] | 79 (15.1%) | 68.3[25.2,100.4] | 5.8[3.0,11.8] | 29 (14.7%) | 24.1[9.7,60.8] |
| DM | 13.5 [6.0,20.6] | 73 (13.9%) | 68.2[23.0,100.4] | 6.7[5.4, 11.1] | 16 (8.1%) | 28.7[13.2,61.9] |
| TSD | 16.4 [9.1,30.9] | 114 (21.8%) | 69.6[28.4,101.1] | 13.4[9.5, 17.6] | 25 (12.7%) | 31.5[14.1,62.0] |
| Death | 25.4 [11.5,51.9] | 234 (44.7%) | 69.6[28.4,101.1] | 14.7[9.8, 23.8] | 42 (21.3%) | 31.5[14.1,62.0] |
| ARD | 17.5 [6.6,46.5] | 248 (47.3%) | 66.4 [20.1, 99.3] | 7.9[5.1, 15.3] | 56 (28.4%) | 23.5[8.7,60.1] |
|  |  |  |  |  |  |  |

LR: local recurrence, RR: regional recurrence, LRR: local or regional recurrence, DM: distant metastasis, TSD: tumor-specific death, ARD: any recurrence or death.

1. **The finally selected features and their coefficients in multivariable clinical and combined models**

The finally selected features and their coefficients in multivariable clinical and combined models (autoencoder, radiomics and end-to-end) are summarized in Table S3. From Table S3, age, HPV-status, WHO PS, T-stage and N-stage were found to be significantly associated with outcome endpoints in clinical models. In all combined models, the linear predictor from the clinical models was significantly associated with all outcomes. For the high-level image features extracted by the autoencoder in the combined autoencoder models, feature number 956_ct (956^th^ feature from the total 1024 features) was found to be a significant predictor of LC, TSS and OS, 567_ct had significant association with LC and LRC and 695_ct was significantly predictive for OS and DFS. In the combined radiomics models, the shape feature-original_shape_LeastAxisLength was a significant predictor of LC, RC and DFS, and the texture feature-original_ngtdm_Busyness was significantly correlated with the prediction of LC, LRC and TSS. In the combined end-to-end models, all predictors of ResNet18 or DenseNet 121 were significantly associated with the prediction of all endpoints. Other image features associated with one outcome were also displayed in Table S3. Additionally, we calculated the Pearson Correlation coefficients between predictors in each model. All the coefficients for clinical, combined autoencoder and combined radiomics models are less than 0.45 and for the combined end-to-end models < 0.62, which indicates that the predictors in the models are not redundant (when using a threshold of 0.70).

Table S3. Estimated coefficients (Coef) of clinical and combined models.

|  |  | | | Clinical models | | |  | Combined autoencoder models | | | | | |  |
| --- | --- | --- | --- | --- | --- | --- | --- | --- | --- | --- | --- | --- | --- | --- |
|  | | Coef | Coef (95% CI) | | HR | P-value | Corr | |  | Coef (95% CI) | | HR | P-value | Corr |
| **Local control (LC)**  Age  HPV-status  LP clinical model  956_ct  567_ct | | 0.07  -1.76 | (0.02, 0.11)  (-2.62, -0.91) | | 1.07  0.17 | <0.005  <0.005 | 0.77  -0.79 | | 0.95  -4.46  2.85 | | (0.59, 1.31)  (-8.94, 0.02)  (0.92, 4.78) | 2.59  0.01  17.29 | < 0.005  0.048  < 0.005 | 0.84  -0.53  0.37 |
| **Regional control (RC)**  N-stage  HPV-status  LP clinical model  614_ct  333_ct  160_ct | | 1.15  -1.69 | (0.24, 2.06)  (-2.64, -0.75) | | 3.16  0.18 | 0.01  < 0.005 | 0.56  -0.72 | | 1.33  3.24  -5.57  -18.85 | | (0.73, 1.93)  (0.27, 6.21)  (-10.02, -1.12)  (-30.68, -7.02) | 3.79  25.53  0.01  0.01 | < 0.005  0.03  0.01  < 0.005 | 0.47  0.13  -0.24  -0.79 |
| **Locoregional control (LRC)**  HPV-status  N-stage  LP clinical model  567_ct  444_ct | | -1.92  0.71 | (-2.65, -1.20)  (0.03, 1.39) | | 0.15  2.04 | < 0.005  0.04 | -0.90  0.28 | | 0.90  2.62  -5.11 | | (0.52, 1.28)  (0.83, 4.41)  (-8.37, -1.85) | 2.45  13.73  0.01 | < 0.005  < 0.005  < 0.005 | 0.68  0.45  -0.71 |
| **Distant metastases free survival (DMFS)**  N-stage  WHO PS  LP clinical model  911_ct  469_ct | | 0.93  0.85 | (0.22, 1.65)  (0.13, 1.56) | | 2.54  2.33 | 0.01  0.02 | 0.73  0.62 | | 0.88  2.46  2.02 | | (0.29, 1.47)  (0.29, 4.62)  (0.42, 3.62) | 2.42  11.65  7.54 | < 0.005  0.03  0.01 | 0.77  0.41  0.64 |
| **Tumor specific survival (TSS)**  HPV-status  N-stage  Age  T-stage  LP clinical model  956_ct_gtv | | -1.32  1.12  0.05  0.86 | (-1.93, -0.70)  (0.49, 1.76)  (0.02, 0.08)  (0.25, 1.47) | | 0.27  3.08  1.05  2.36 | < 0.005  < 0.005  < 0.005  0.01 | -0.57  0.42  0.55  0.38 | | 0.96  -4.41 | | (0.66, 0.26)  (-7.64, -1.18) | 2.60  0.01 | < 0.005  0.01 | 0.87  -0.59 |
| **Overall Survival (OS)**  Age  HPV-status  WHO PS  T-stage  N-stage  LP clinical model  695_ct  517_ct  956_ct | | 0.04  -1.02  0.58  0.58  0.48 | (0.02, 0.06)  (-1.48, -0.57)  (0.15, 0.01)  (0.15, 1.01)  (0.06, 0.90) | | 1.04  0.36  1.78  1.79  1.62 | < 0.005  < 0.005  0.01  0.01  0.02 | 0.61  -0.65  0.58  0.37  0.12 | | 0.88  1.40  1.90  -2.06 | | (0.60, 1.15)  (0.26, 2.54)  (0.47, 3.34)  (-3.86, -0.26) | 2.40  4.06  6.72  0.13 | < 0.005  0.02  0.01  0.03 | 0.87  0.57  0.36  -0.42 |
| **Disease free survival (DFS)**  HPV-status  Age  WHO PS  T-stage  LP clinical model  220_ct  695_ct | | -0.86  0.03  0.55  0.50 | (-1.29, -0.44)  (0.01, 0.05)  (0.13, 0.96)  (0.07, 0.93) | | 0.42  1.03  1.73  1.65 | < 0.005  < 0.005  0.01  0.02 | -0.68  0.65  0.62  0.35 | | 0.87  -3.32  1.16 | | (0.57, 1.17)  (-5.09, -1.54)  (0.01, 2.31) | 2.38  0.04  3.19 | < 0.005  < 0.005  0.047 | 0.85  -0.64  0.62 |

WHO PS: World Health Organization performance status, Coef: coefficients, CI: confidence interval, HR: hazard ratio, LP: linear predictor, Corr: correlation coefficient between feature and the finial LP of the model. The reference groups for HPV-status, N-stage, WHO PS and T-stage are (HPV-status = negative), (N-stage = N0-N1), (WHO PS = 0) and (T-stage = T1-T3), respectively.

| Combined radiomics models | | | | |  |
| --- | --- | --- | --- | --- | --- |
|  | Coef | Coef (95% CI) | HR | P-value | Corr |
| **Local control (LC)**  LP clinical model  original_ngtdm_Busyness  original_shape_LeastAxisLength | 1.05  0.16  0.08 | (0.67, 1.43)  (0.07, 0.25)  (0.03, 0.13) | 2.85  1.17  1.08 | <0.005  <0.005  <0.005 | 0.85  0.64  0.62 |
| **Regional control (RC)**  LP clinical model  original_shape_LeastAxisLength | 0.96  0.09 | (0.47, 1.45)  (0.03, 0.14) | 2.62  1.09 | < 0.005  < 0.005 | 0.77  0.74 |
| **Locoregional control (LRC)**  LP clinical model  original_ngtdm_Busyness | 1.07  0.16 | (0.69, 1.45)  (0.07, 0.24) | 2.92  1.17 | < 0.005  < 0.005 | 0.95  0.27 |
| **Distant metastases free survival (DMFS)**  LP clinical model  original_shape_MinorAxisLength | 0.90  0.03 | (0.33, 1.48)  (0.00, 0.06) | 2.47  1.03 | <0.005  0.05 | 0.87  0.66 |
| **Tumor specific survival (TSS)**  LP clinical model  Original_ngtdm_Busyness | 1.05  0.14 | (0.74, 1.35)  (0.06, 0.21) | 2.84  1.15 | < 0.005  < 0.005 | 0.97  0.27 |
| **Overall Survival (OS)**  LP clinical model  Original_shape_Maximum2DDiameterSlice | 0.89  0.01 | (0.61, 1.17)  (0.00, 0.03) | 2.44  0.01 | < 0.005  0.09 | 0.98  0.61 |
| **Disease free survival (DFS)**  LP clinical model  original_shape_LeastAxisLength | 0.86  0.05 | (0.57, 1.15)  (0.02, 0.07) | 2.36  1.05 | < 0.005  < 0.005 | 0.89  0.76 |

|  | Combined models (ResNet18) | | | |  | | | Combined models (DenseNet121) | | | | | |  |
| --- | --- | --- | --- | --- | --- | --- | --- | --- | --- | --- | --- | --- | --- | --- |
|  | Coef | Coef (95% CI) | HR | P-value | Corr |  | | | Coef Coef (95% CI) | | HR | | P-value | Corr |
| **Local control (LC)**  LP clinical model  LP ResNet18  LP DenseNet121 | 0.78  1.73 | (0.40, 1.15)  (0.98, 2.49) | 2.18  5.67 | <0.005  <0.005 | 0.81  0.87 | | 0.76  1.95 | | | (0.38, 1.14)  (1.10, 2.80) | | 2.14  7.03 | <0.005  <0.005 | 0.78  0.90 |
| **Regional control (RC)**  LP clinical model  LP ResNet18  LP DenseNet121 | 0.80  1.39 | (0.28, 1.33)  (0.72, 2.06) | 2.23  4.00 | <0.005  <0.005 | 0.72  0.87 | | 0.86  1.03 | | | (0.34, 1.39)  (0.48, 1.59) | | 2.37  2.81 | <0.005  <0.005 | 0.78  0.82 |
| **Locoregional control (LRC)**  LP clinical model  LP ResNet18  LP DenseNet121 | 0.75  1.37 | (0.37, 1.13)  (0.81, 1.93) | 2.11  3.93 | <0.005  <0.005 | 0.75  0.87 | | 0.62  1.85 | | | (0.23, 1.00)  (1.21, 2.48) | | 1.85  6.34 | <0.005  <0.005 | 0.67  0.94 |
| **Distant metastases free survival (DMFS)**  LP clinical model  LP ResNet18  LP DenseNet121 | 0.93  1.72 | (0.33, 1.53)  (1.03, 2.42) | 2.53  5.61 | <0.005  <0.005 | 0.65  0.88 | | 0.83  1.38 | | | (0.24, 1.41)  (0.79, 1.97) | | 2.29  3.98 | 0.01  <0.005 | 0.67  0.88 |
| **Tumor specific survival (TSS)**  LP clinical model  LP ResNet18  LP DenseNet121 | 0.60  1.64 | (0.24, 0.95)  (0.89, 2.39) | 1.82  5.14 | <0.005  <0.005 | 0.84  0.91 | | 0.66  1.52 | | | (0.31, 1.01)  (0.80, 2.24) | | 1.94  4.59 | <0.005  <0.005 | 0.85  0.89 |
| **Overall Survival (OS)**  LP clinical model  LP ResNet18  LP DenseNet121 | 0.58  1.46 | (0.28, 0.88)  (0.91, 2.00) | 1.78  1.30 | <0.005  <0.005 | 0.85  0.93 | | 0.60  1.13 | | | (0.29, 0.91)  (0.64, 1.62) | | 1.82  3.10 | <0.005  <0.005 | 0.88  0.92 |
| **Disease free survival (DFS)**  LP clinical model  LP ResNet18  LP DenseNet121 | 0.47  1.91 | (0.15, 0.80)  (1.33, 2.48) | 1.61  6.74 | <0.005  <0.005 | 0.79  0.97 | | 0.51  1.36 | | | (0.18, 0.83)  (0.90, 1.81) | | 1.66  3.89 | <0.005  <0.005 | 0.81  0.96 |

Table S4. The C-index results of combined end-to-end models based on either RseNet18 or DenseNet121 in the training, internal and external test sets.

|  | Training set |  | Internal test set |  | External test set |  |
| --- | --- | --- | --- | --- | --- | --- |
|  | Combined model (ResNet18) | Combined model (DenseNet121) | Combined model (ResNet18) | Combined model (DenseNet121) | Combined model (ResNet18) | Combined model (DenseNet121) |
| LC | **0.88[0.81, 0.94]*** | **0.89[0.83, 0.94]*** | **0.82[0.67, 0.94]*** | 0.74[0.56, 0.89] | 0.75[0.65 0.85] | 0.75[0.64, 0.85] |
| RC | 0.85[0.77, 0.92] | 0.83[0.73, 0.92] | 0.83[0.64, 0.96] | 0.84[0.62, 0.99] | 0.69[0.57, 0.81] | 0.70[0.56, 0.81] |
| LRC | **0.82[0.74, 0.89]*** | **0.86[0.80, 0.92]*** | **0.79[0.65, 0.91]*** | 0.77[0.61, 0.89] | 0.72[0.63,0.81] | 0.71[0.62, 0.80] |
| DMFS | **0.79[0.70, 0.87]** | **0.79[0.70,0.87]*** | 0.64[0.50, 0.77] | 0.68[0.57, 0.79] | 0.72[0.56, 0.86] | 0.72[0.58, 0.84] |
| TSS | **0.80[0.73, 0.86]*** | **0.80[0.72, 0.86]*** | 0.69[0.57, 0.79] | **0.72[0.60, 0.81]** | **0.80[0.71, 0.88]*** | **0.80[0.72, 0.87]*** |
| OS | **0.77[0.72, 0.82]*** | **0.76[0.71, 0.82]** | 0.71[0.63, 0.78] | **0.73[0.66, 0.79]*** | 0.78[0.72, 0.84] | 0.78[0.72, 0.85] |
| DFS | **0.76[0.71, 0.81]*** | **0.76[0.70, 0.81]*** | 0.72[0.65, 0.79] | 0.73[0.66, 0.79] | 0.69[0.62, 0.77] | 0.70[0.64, 0.78] |

The underlined C-indexes are slightly higher (C-index improvement < 0.01) than that of all other models in Table 1. *: Significant difference of C-indexes between the combined model (ResNet18 or DenseNer121) and all other models in Table 1 (p-Value < 0.05 by z-test).

1. **Test for HPV positive and HPV negative patients, respectively**

We further investigated the performance of clinical, combined autoencoder and combined radiomics models for HPV-positive and HPV-negative patients separately(Table S5). In the internal test set, combined autoencoder models achieved highest C-index values in most endpoints for both HPV-positive and HPV-negative patients. In the external test set, combined radiomics models displayed best predictive performance for all endpoints except TSS of HPV-positive patients.

Table S5. The C-index results of clinical models, combined autoencoder models, combined radiomics models in the internal and external test sets for HPV positive and HPV negative patients, respectively.

|  |  |  | Internal test set | | |  | External test set | | |
| --- | --- | --- | --- | --- | --- | --- | --- | --- | --- |
| Patients | Endpoint | Event number (percentage) | Clinical model | Combined  autoencoder  model | Combined  radiomics  model | Event number | Clinical model | Combined  autoencoder  model | Combined  radiomics model |
|  | LC | 4 (4%) | 0.64 | **0.67** | 0.65 | 4 (4%) | 0.33 | 0.65 | **0.84** |
|  | RC | 2 (2%) | 0.55 | **0.90** | 0.73 | 5 (5%) | 0.52 | 0.50 | **0.82** |
|  | LRC | 5 (5%) | 0.55 | 0.70 | **0.71** | 7 (7%) | 0.53 | 0.70 | **0.72** |
| HPV positive | DMFS | 13 (14%) | 0.61 | **0.76** | 0.67 | 5 (5%) | 0.66 | 0.61 | **0.67** |
|  | TSS | 14 (15%) | 0.64 | **0.65** | 0.63 | 6 (6%) | 0.64 | **0.69** | 0.66 |
|  | OS | 23 (24%) | 0.64 | **0.68** | 0.65 | 9 (9%) | 0.75 | 0.79 | **0.80** |
|  | DFS | 25 (26%) | 0.68 | **0.68** | 0.68 | 15 (15%) | 0.63 | 0.65 | **0.76** |
|  | LC | 8 (17%) | 0.51 | 0.75 | **0.82** | 13 (14%) | 0.66 | 0.67 | **0.76** |
|  | RC | 4 (9%) | 0.71 | **0.86** | 0.76 | 15 (16%) | **0.65** | 0.58 | 0.62 |
|  | LRC | 10 (22%) | 0.51 | **0.66** | 0.60 | 22 (23%) | 0.60 | 0.60 | **0.63** |
| HPV negative | DMFS | 7 (15%) | 0.58 | **0.71** | 0.66 | 11 (12%) | 0.69 | 0.65 | **0.70** |
|  | TSS | 14 (30%) | 0.61 | **0.65** | 0.62 | 19 (20%) | 0.72 | 0.71 | **0.76** |
|  | OS | 30 (65%) | 0.58 | 0.58 | **0.61** | 33 (35%) | 0.72 | 0.72 | **0.74** |
|  | DFS | 32 (70%) | 0.60 | 0.62 | **0.63** | 41 (43%) | 0.62 | 0.66 | **0.68** |

The underlined C-indexes are slightly higher (C-index improvement < 0.01) than that of the other model in the same dataset.

1. **Description of the calibration curves of combined radiomics models**

The predicted outcome rate curves of the combined radiomics models predicted showed good overlap (within the 95% CIs) with the actual KM curves for LC, RC, DMFS, TSS, OS and DFS in the internal test set and for LC, RC, LRC, DMFS OS, and DFS in the external test set within 2-year (Figure S4A). Additionally, combined radiomics models showed good calibration (p > 0.05 by HS test) for 2-year LC, OS and DFS in the internal test set and for all endpoints except RC in the external test set (Figure S5B). Good calibration lines were obtained for OS in the internal test set and RC, LRC, TSS, OS and DFS in the external test set.

For LC and OS, combined radiomics models achieved good discriminative and calibration abilities with higher C-indexes of 0.82 and 0.81 in the external test set than the combined autoencoder models (Table 1). However, the combined radiomics models showed poor calibration (p < 0.05) for more endpoints (2-year RC, LRC, DMFS, TSS) in the internal test set (Figure S4B), which further demonstrated that radiomics is less effective in extracting representative features than the autoencoders for the internal test.

1. **More discussion**

We found that tumor volume, one radiomics feature, is a significant predictor (univariable Cox analysis) for all endpoints in the training set. However, it was not selected in the final combined radiomics. The tumor volume was highly correlated with all selected shape radiomics features (correlation coefficients of 0.87, 0.80 and 0.84 for original_shape_LeastAxisLength, original_shape_MinorAxisLength and original_shape_Maximum2DDiameterSlice, respectively) in our combined radiomics models in Table S3. For combined autoencoder models, tumor volume only showed a moderate correlation with all features (correlation coefficient < 0.40 except 0.60 for 695_ct_gtv). Additionally, the tumor volume has correlation coefficients < 0.60 with all linear predictors of combined radiomics or autoencoder models .

This study compared the predictive performance of CT image features extracted by radiomics, self-supervised learning and end-to-end deep learning, which are common methods used for outcome prediction of HNC [6]. For example, Starke et. al. compared end-to-end deep learning, transfer-learning and deep autoencoder features for LRC prediction in a multicenter cohorts but did not include radiomics features [7]. They found that 3D end-to-end model achieved highest C-index of 0.69 in the independent validation cohort. Our combined end-to-end model also obtained highest C-index of 0.79 in the internal test set (Table S4) while combined radiomics model predicted best with C-index of 0.75 in the external test set. Chen et. al. adopted pre-trained 3D video classification network to extract CT image features for outcome prediction [8]. Although their deep features outperformed radiomics features in OS prediction of HNC patients (C-index: 0.76 vs. 0.73) in the internal test set, radiomics features showed better external validation and higher reproducibility. Our conclusion that autoencoder-extracted deep features outperformed radiomics features only in the internal test cohort and not in the external test is in agreement with their finding [8].

1. **limitations**

Some limitations exist in this study. Firstly, the percentage of events in the training set were low (Table S1). Especially for RR with an event rate of 7.1%, which may restrict prediction performance of the built models. Secondly, we did not extract image features from the pathological lymph nodes which probably contain more predictive image information than the primary tumor for RC and DMFS prediction. Finally, the interpretability of autoencoder and end-to-end extracted features is limited. Although technologies like GradCam [9] can locate the highly contributing image regions, the clinical meanings of features from those regions should be further explored. The radiomics features can be interpreted easier due to their clear definitions. In the future, standardization of CT acquisition and tumor delineation between centers, training models using multi-modality images around primary tumors and pathological lymph nodes, training prediction models with more patients from multi-centers with more events may improve the prediction performance and the generalizability of our models especially for deep learning models.

Figure S2. Kaplan-Meier curves of high (hazard values > median) and low (hazard values <= median) risk groups of LC, RC, LRC, DMFS, TSS, OS and DFS on the independent internal and external test sets by clinical (A) ,combined autoencoder models (B) and combined radiomics models (C).


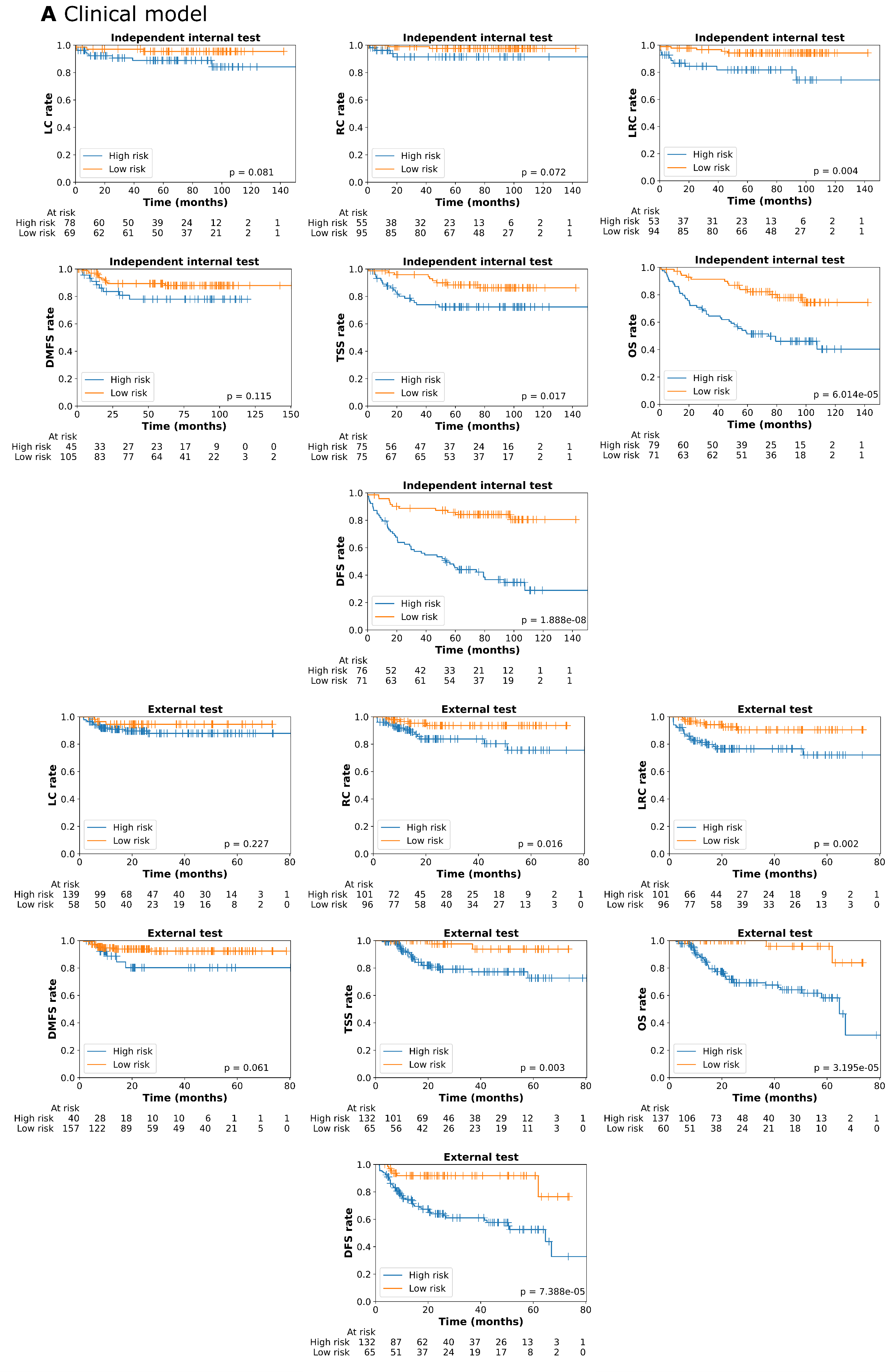


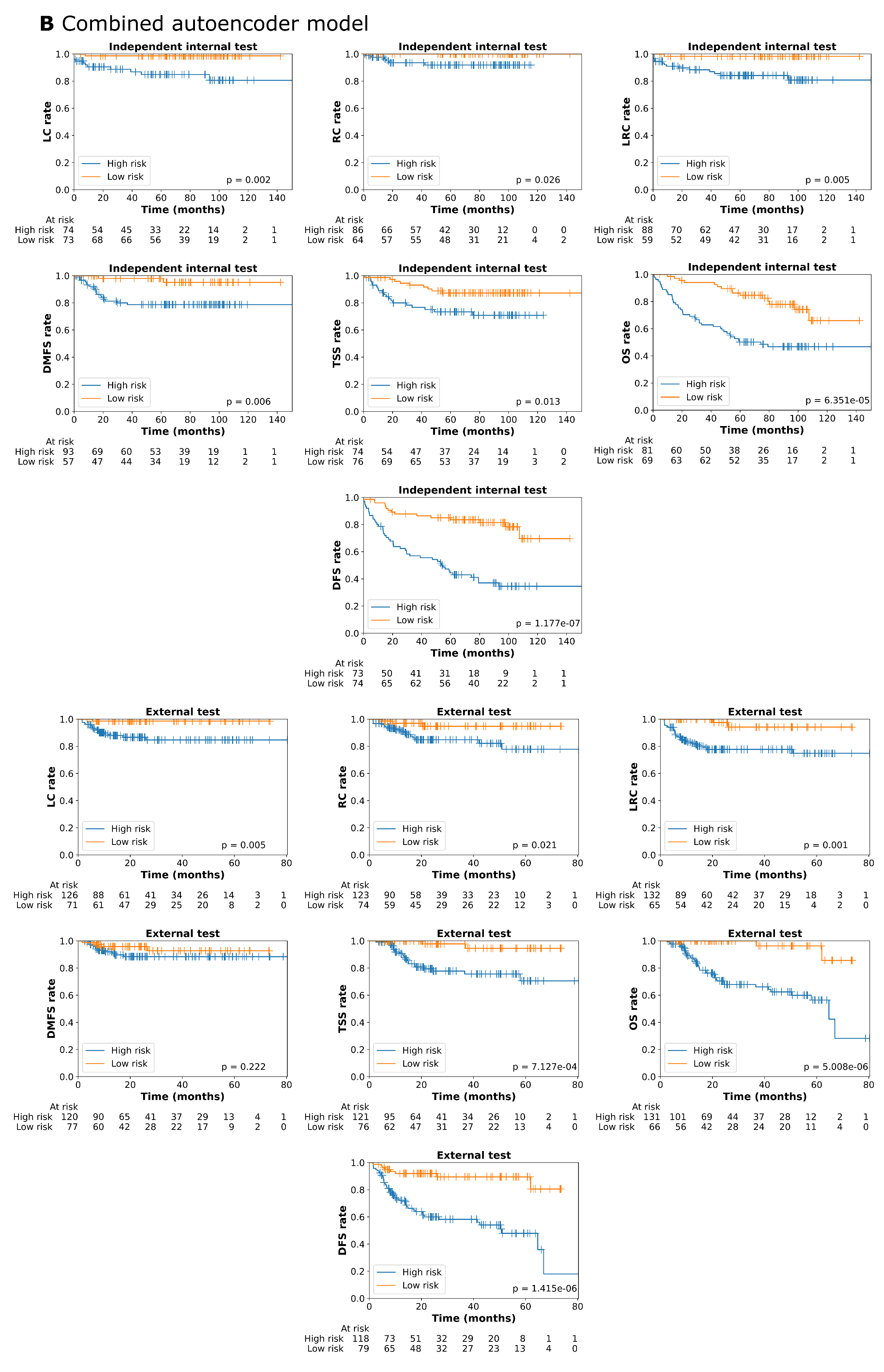


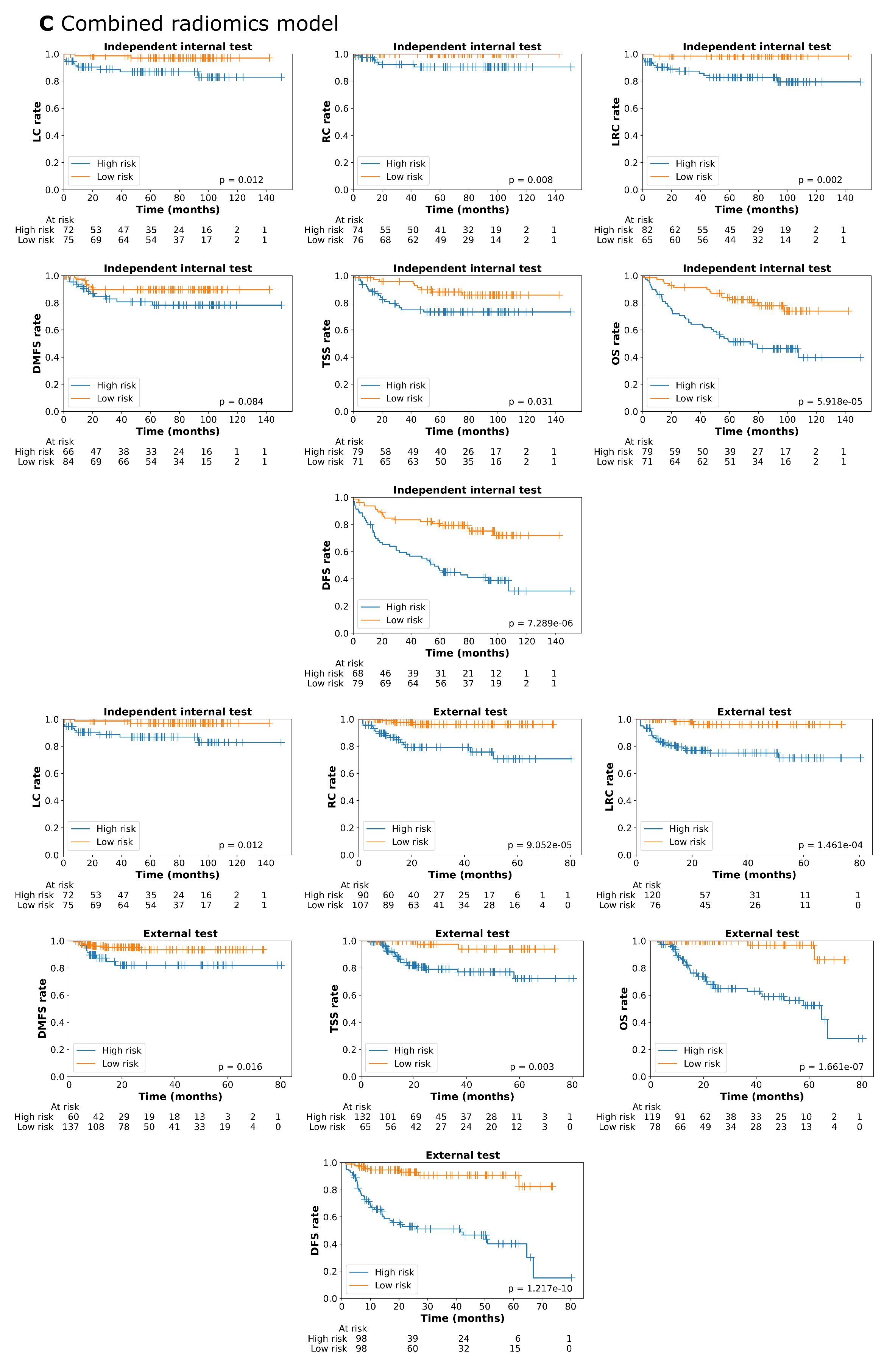


Figure S3. Calibration plots of combined autoencoder models for LC, RC, LRC, DMFS, TSS, OS and DFS on the independent internal and external test sets in the (A) within 5-year (B) at 2-year. p-Values were from HS tests . Slope and intercept belong to real calibration line.


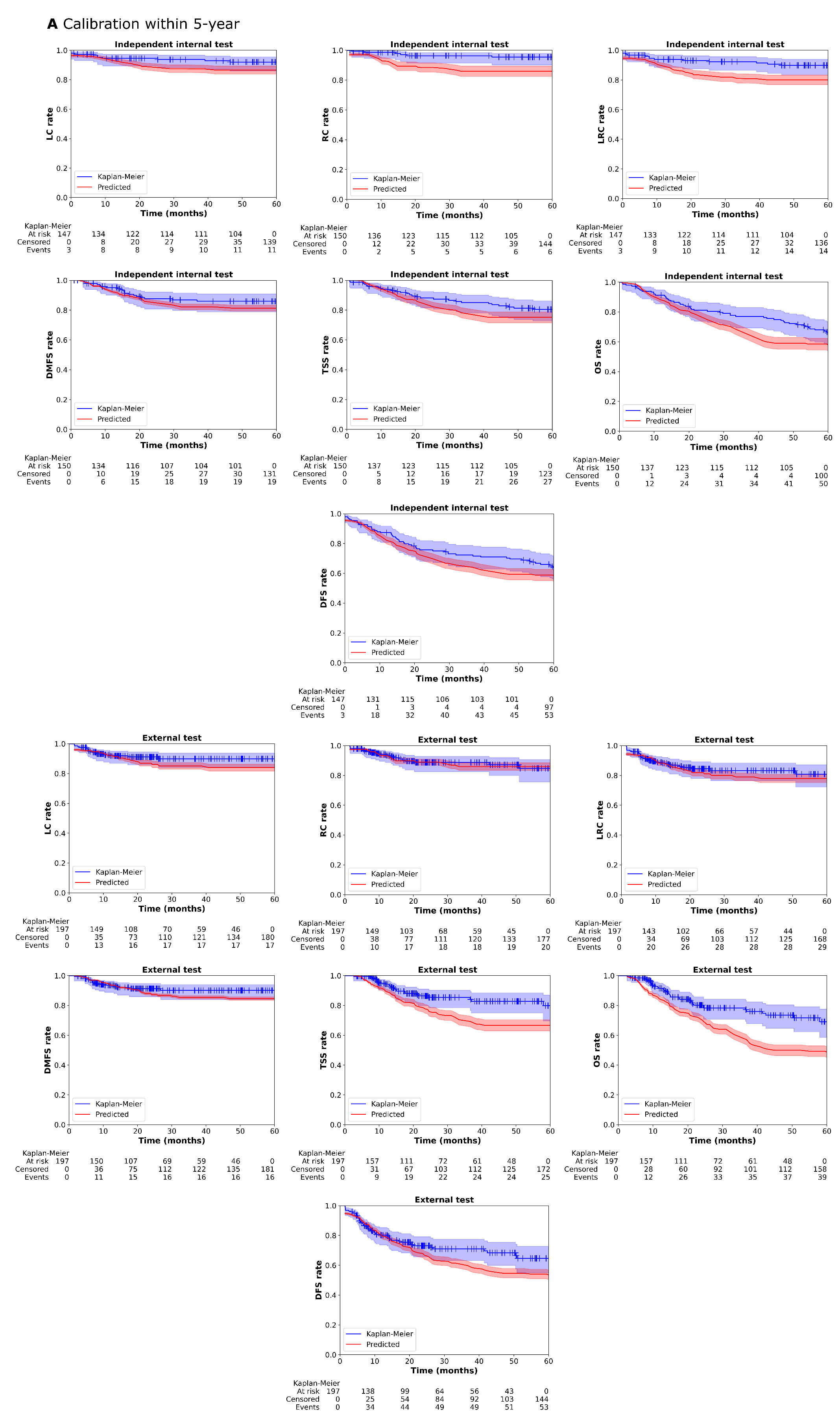


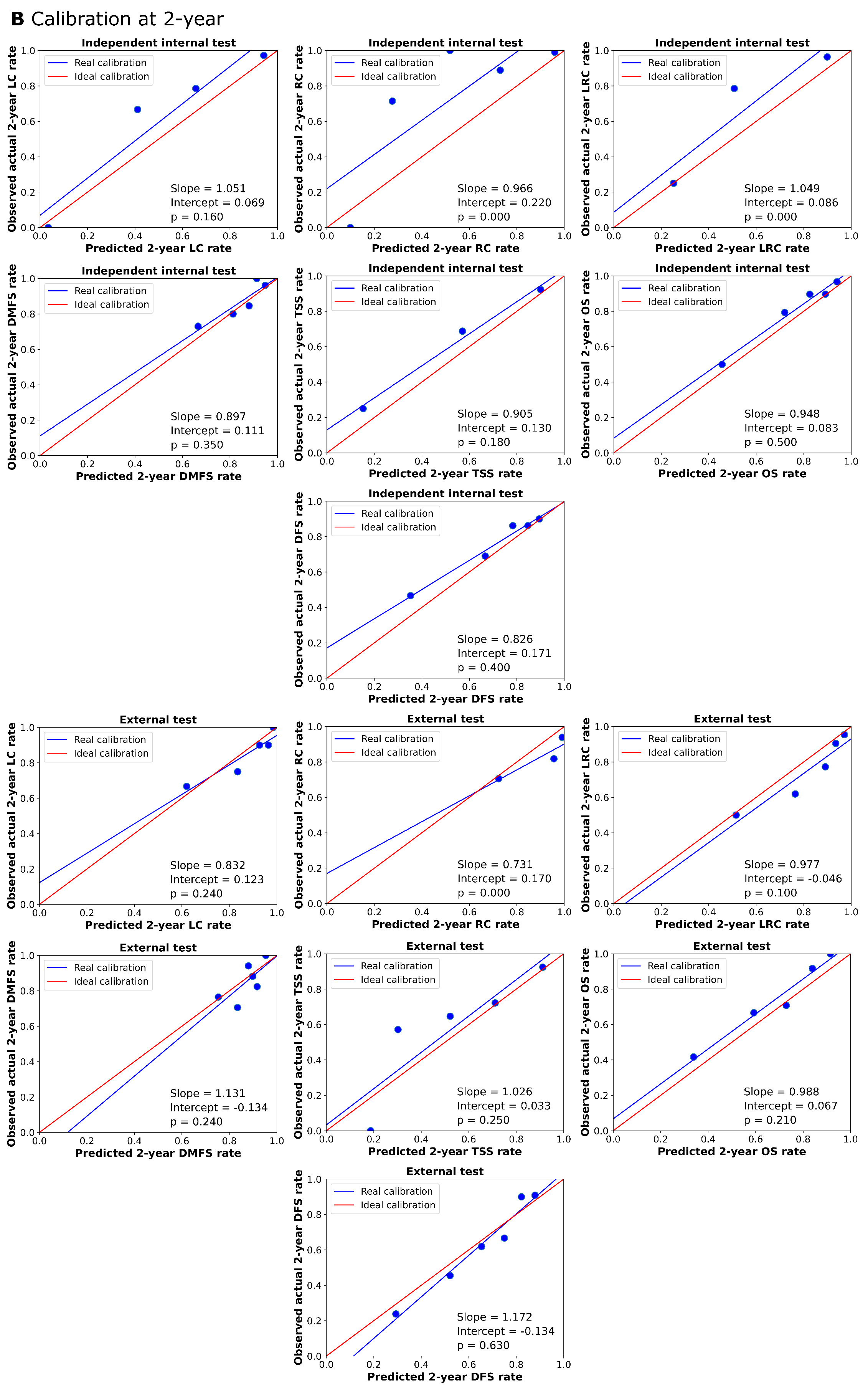


Figure S4. Calibration plots of combined radiomics models for LC, RC, LRC, DMFS, TSS, OS and DFS on the independent internal and external test sets in the (A) within 5-year (B) at 2-year. p-Values were from HS tests . Slope and intercept belong to real calibration line.


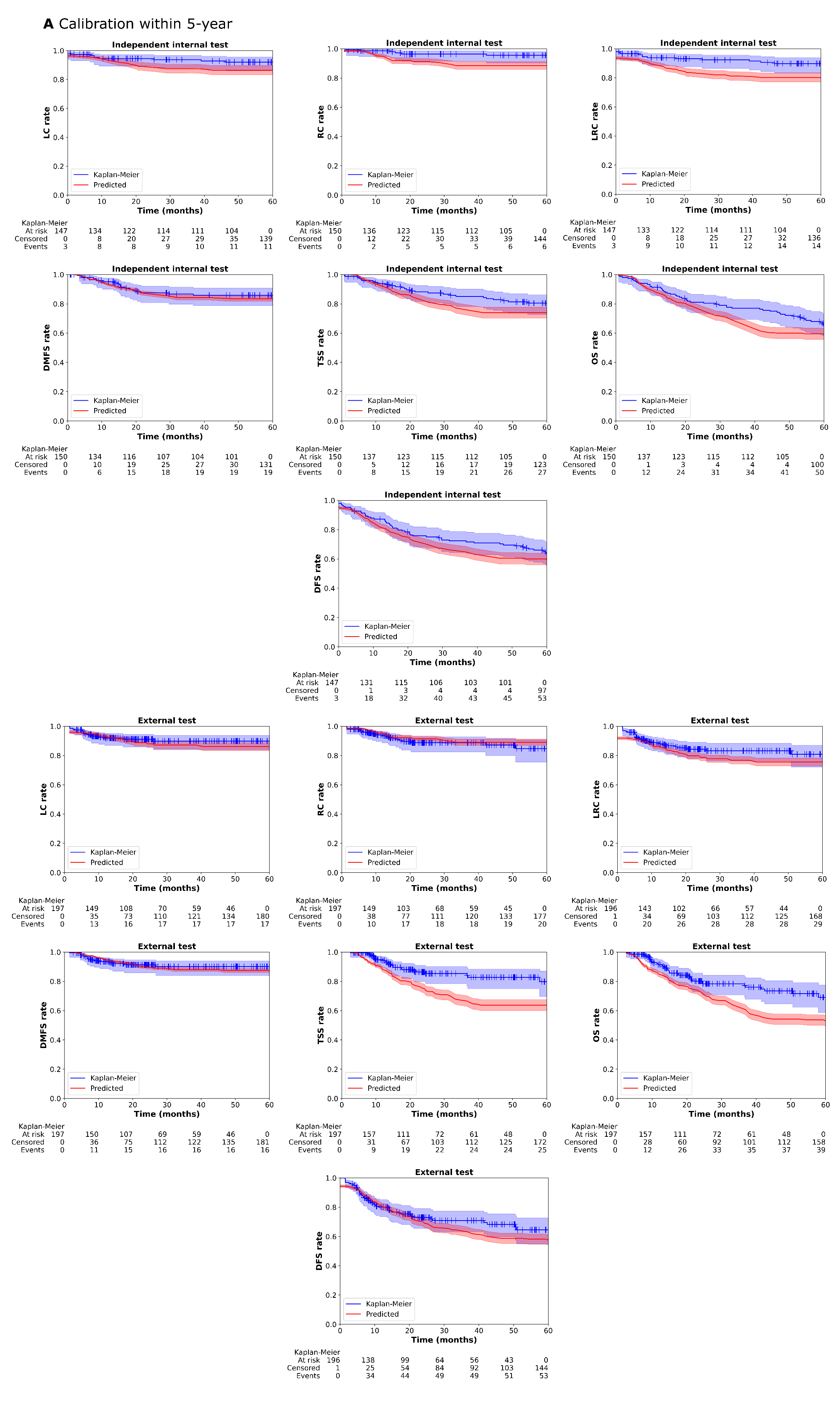


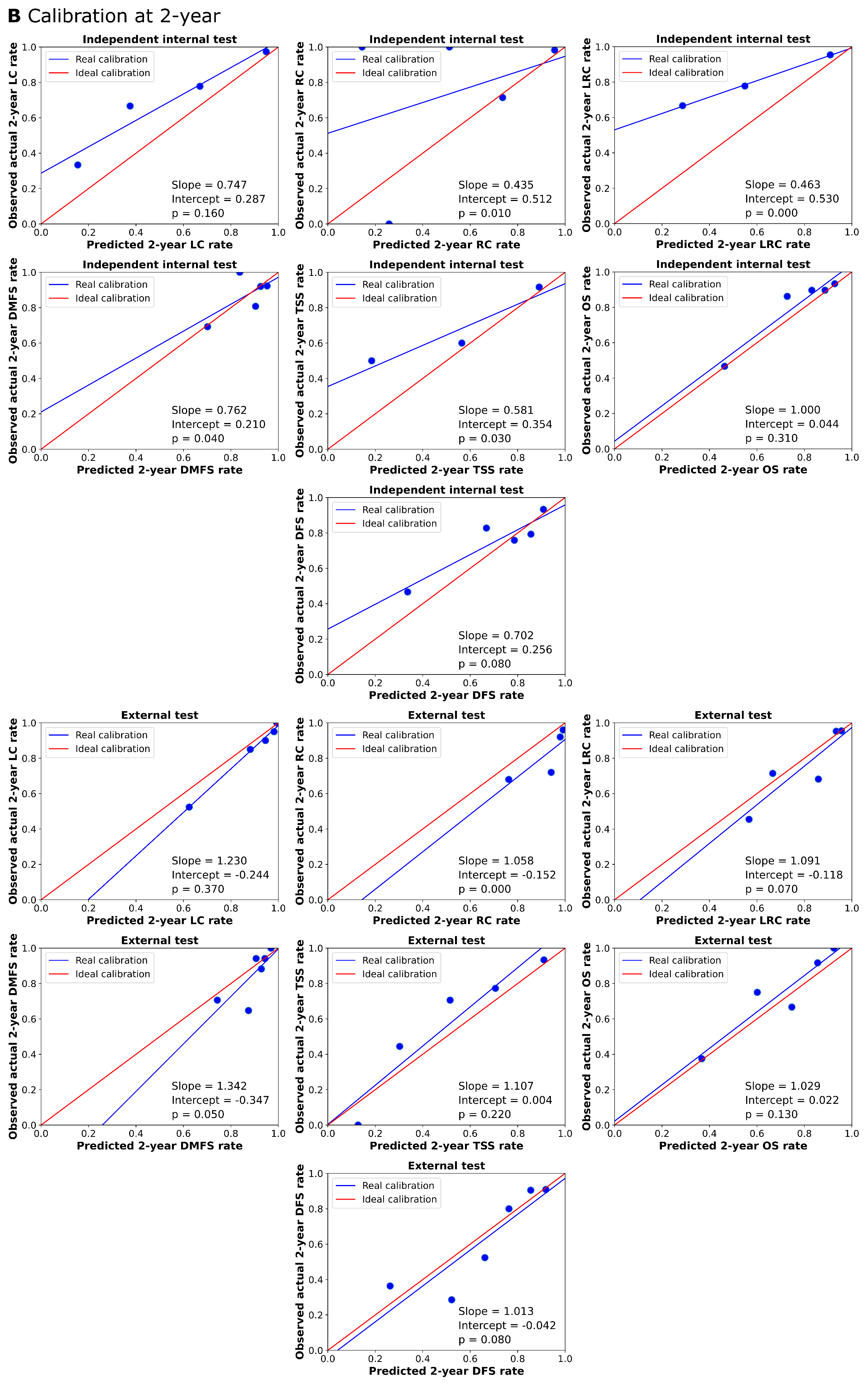


**Reference**

[1] Wang Z, Bovik AC, Sheikh HR, Simoncelli EP. Image quality assessment: From error visibility to structural similarity. IEEE Trans Image Process 2004;13. https://doi.org/10.1109/TIP.2003.819861.

[2] He K, Zhang X, Ren S, Sun J. Deep residual learning for image recognition. Proc. IEEE Comput. Soc. Conf. Comput. Vis. Pattern Recognit., vol. 2016- December, 2016. https://doi.org/10.1109/CVPR.2016.90.

[3] Huang G, Liu Z, Van Der Maaten L, Weinberger KQ. Densely connected convolutional networks. Proc. IEEE Conf. Comput. Vis. pattern Recognit., 2017, p. 4700–8.

[4] Katzman JL, Shaham U, Cloninger A, Bates J, Jiang T, Kluger Y. DeepSurv: Personalized treatment recommender system using a Cox proportional hazards deep neural network. BMC Med Res Methodol 2018;18. https://doi.org/10.1186/s12874-018-0482-1.

[5] Ma B, Guo J, Van Dijk L, van Ooijen PMA, Both S, Sijtsema NM. TransRP: Transformer-based PET/CT feature extraction incorporating clinical data for recurrence-free survival prediction in oropharyngeal cancer. Med. Imaging with Deep Learn., 2023.

[6] Mahmood H, Shaban M, Rajpoot N, Khurram SA. Artificial Intelligence-based methods in head and neck cancer diagnosis: an overview. Br J Cancer 2021;124:1934–40.

[7] Starke S, Leger S, Zwanenburg A, Leger K, Lohaus F, Linge A, et al. 2D and 3D convolutional neural networks for outcome modelling of locally advanced head and neck squamous cell carcinoma. Sci Rep 2020;10. https://doi.org/10.1038/s41598-020-70542-9.

[8] Chen J, Wee L, Dekker A, Bermejo I. Using 3D deep features from CT scans for cancer prognosis based on a video classification model: A multi‐dataset feasibility study. Med Phys 2023.

[9] Selvaraju RR, Cogswell M, Das A, Vedantam R, Parikh D, Batra D. Grad-CAM: Visual Explanations from Deep Networks via Gradient-Based Localization. Int J Comput Vis 2020;128. https://doi.org/10.1007/s11263-019-01228-7.
